# A label-free impedimetric immunosensor based on nitrogen-doped graphene acid for sensitive detection of vitamin D_3_

**DOI:** 10.1007/s00604-025-07625-9

**Published:** 2025-11-18

**Authors:** Jakub Janek, Zdenka Fohlerova, Ivan Dědek, Vítězslav Hrubý, David Panáček, Jaromir Hubalek, Roman Havlík, Radek Zbořil, Michal Otyepka, Petr Jakubec

**Affiliations:** 1 Department of Microelectronics, Faculty of Electrical Engineering and Communication, Brno University of Technology, Technická 10, Brno, 616 00 Czech Republic; 2 Department of Biomedical Engineering, Faculty of Electrical Engineering and Communication, Brno University of Technology, Technická 10, Brno, 616 00 Czech Republic; 3 Regional Centre of Advanced Technologies and Materials (RCPTM), Czech Advanced Technology and Research Institute (CATRIN), Palacký University Olomouc, Šlechtitelů 27, Olomouc, 783 71 Czech Republic; 4 Nanotechnology Centre, Centre for Energy and Environmental Technologies, VSB–Technical University of Ostrava, 17. Listopadu 2172/15, Ostrava-Poruba, 708 00 Czech Republic; 5 Department of Surgery I, Faculty of Medicine and Dentistry, Palacký University Olomouc and University Hospital Olomouc, Olomouc, 771 47 Czech Republic; 6 IT4Innovations, VSB – Technical University of Ostrava, 17. Listopadu 2172/15, Ostrava-Poruba, 708 00 Czech Republic

**Keywords:** 25-Hydroxyvitamin D_3_, Electrochemical detection, Impedance measurement, Immunosensor, Nitrogen-doped graphene acid, Metal-free

## Abstract

A simple metal-free electrochemical immunosensor based on nitrogen-doped graphene acid (NGA) for the detection of 25-hydroxy vitamin D_3_ (25-OH D_3_) is presented. The use of NGA, with its well-defined chemical composition, high density of carboxyl functional groups, and excellent biocompatibility, provided a stable and efficient platform for biomolecule immobilization without the need for complex surface modifications. The proposed immunosensor demonstrated high sensitivity (1.97 kΩ ng^‒1^ mL cm^‒2^) and a working range of 3.96 to 48.83 ng mL^‒1^, aligning with clinically relevant vitamin D levels. Moreover, the developed immunosensor exhibited excellent specificity, with minimal cross-reactivity and strong resistance to non-specific adsorption. Furthermore, it maintained stable electrochemical performance for up to 30 days, demonstrating its suitability for practical applications. Compared to existing vitamin D sensors, our platform introduces a streamlined fabrication process that avoids heavy metals, improves reproducibility, and enhances accessibility for point-of-care diagnostics.

**Graphical abstract:**

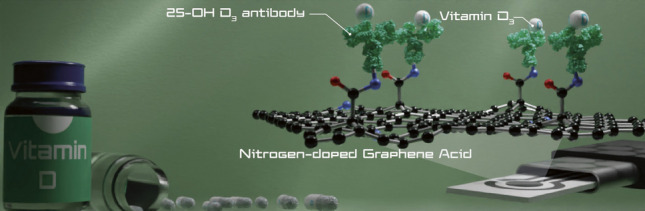

**Supplementary information:**

The online version contains supplementary material available at 10.1007/s00604-025-07625-9.

## Introduction

Vitamin D deficiency is a widespread global health issue, affecting an estimated one billion people worldwide [1]. This deficiency is particularly prevalent among populations with limited sun exposure, including children, the elderly, and individuals living in northern latitudes [2]. Vitamin D_3_ plays a critical role in calcium metabolism, regulating calcium and phosphorus homeostasis, which is essential for maintaining healthy bones [3]. Deficiency in vitamin D_3_ has been linked to conditions such as osteoporosis in adults and rickets in children. Additionally, emerging evidence suggests that low vitamin D levels may contribute to increased susceptibility to infections, including respiratory illnesses, and could be associated with a higher risk of autoimmune disorders [4]. These findings highlight the importance of accurately monitoring vitamin D levels to prevent and manage deficiency-related health complications.

The standard clinical biomarker for assessing vitamin D status is 25-OH D_3_. Blood serum levels of 25-OH D_3_ are considered insufficient when ranging between 20 and 30 ng mL^‒1^ and deficient when falling below 20 ng mL^‒1^ [5]. Given these thresholds, highly sensitive and precise detection methods are crucial for ensuring accurate monitoring and early intervention in vitamin D deficiency. The most widely used methods for measuring serum 25-OH D_3_ concentrations include immunoassays, such as enzyme-linked immunosorbent assay (ELISA), radioimmunoassay (RIA), and chemiluminescent immunoassay (CLIA), as well as liquid chromatography coupled with mass spectrometry (LC–MS/MS) [6–9]. While immunoassays provide a relatively straightforward and cost-effective approach, they often suffer from limitations such as low sensitivity, high variability, long assay times, and the requirement for large sample volumes [10]. LC–MS/MS offers superior sensitivity and specificity, with the ability to distinguish between different forms of vitamin D. However, its widespread adoption is hindered by high costs, the need for specialized equipment, and labor-intensive sample preparation [11–14]. These limitations highlight the demand for alternative detection strategies that are both reliable, affordable, and accessible.

Electrochemical immunosensors offer a compelling approach due to their simplicity, high sensitivity, miniaturization, and suitability for real-time analysis [15–17]. These sensors utilize the antigen–antibody binding principle, enabling cost-effective, portable, and rapid detection of analytes while maintaining high specificity and sensitivity [18]. Their integration with compact and user-friendly potentiostats makes them particularly attractive for point-of-care (POC) diagnostics [19]. For POC applications, however, further design considerations are critical. Wash-free detection strategies are highly desirable as they eliminate laborious washing steps, shorten assay time, and improve ease of use, which is essential for on-site testing [20–22]. In parallel, effective signal amplification approaches—such as redox cycling—can substantially improve assay sensitivity, thereby enabling detection at clinically relevant levels with minimal instrumentation [23]. Beyond current designs, next-generation nanobiosensor devices are being developed to address the increasing demand for rapid, accurate, and user-friendly diagnostics. These platforms integrate advanced nanomaterials with innovative transduction schemes to achieve higher sensitivity and reproducibility while remaining suitable for decentralized healthcare settings [24–26].

To enhance the performance of electrochemical immunosensors, researchers have explored the incorporation of nanomaterials, particularly carbon-based and metal-derived materials, which improve electron transfer properties and increase the immobilization capacity for biorecognition elements [27, 28]. Among carbon-based nanomaterials, graphene and its derivatives, including reduced graphene oxide (rGO) and carbon nanotubes (CNTs), have attracted attention due to their high electrochemical activity, conductivity, and biocompatibility [29–31].

Despite the promising attributes of electrochemical detection strategies, reported immunosensors for vitamin D_3_ detection involve complex multistep synthesis processes, requiring heavy metals or intricate material modifications [32–34]. These factors call for the development of efficient, eco-friendly, and sustainable approaches in the design of respective electrochemical immunosensors. Graphene, rGO, and CNTs are among the most studied carbon-based nanomaterials. [29, 30, 35]. However, the difficulty in controlling the degree of functionalization of CNT and rGO, along with limited colloidal stability and uniformity of the functional groups, makes both nanomaterials less suitable for bioaffinity assays, which require highly reproducible specific bioconjugation reactions. Graphene acid (GA) has emerged as a highly efficient and reproducible platform for biosensor applications due to its hydrophilicity, high conductivity, and the presence of uniformly distributed carboxyl functional groups, facilitating efficient biomolecule immobilization [36, 37]. NGA has been developed as a next-generation graphene derivative for biosensing, featuring both out-of-plane carboxyl groups and in-plane nitrogen heteroatoms, which enhance its potential for the construction of biosensors. In addition, NGA is a biocompatible, well-defined 2D material with a high density of carboxyl groups (~ 15 at.%), which enhances its capacity to immobilize biomolecules [38–41].

In this study, we present a novel metal-free electrochemical immunosensor based on NGA-modified screen-printed carbon electrodes (SPCE) for the detection of 25-OH D_3_. The proposed immunosensor demonstrates a sensitivity of 1.97 kΩ ng^‒1^ mL cm^‒2^ and a working range of 3.96 to 48.83 ng mL^‒1^, aligning with clinically relevant vitamin D levels. The incorporation of NGA offers a simplified, cost-effective alternative to traditional methods, with the potential to improve POC diagnostics, facilitate timely medical interventions, and enhance patient outcomes related to vitamin D deficiency.

## Materials and methods

### Chemicals

Potassium hexacyanoferrate(II) trihydrate (K_4_[Fe(CN)_6_]), potassium hexacyanoferrate(III) (K_3_[Fe(CN)_6_]), phosphate buffer saline (PBS; 10 mmol L^−1^, pH 7.4), N-(3-dimethylaminopropyl)-N′-ethylcarbodiimide hydrochloride (EDC), *N*-hydroxysuccinimide (NHS), bovine serum albumin (BSA), 2-(N-Morpholino)ethanesulfonic acid (MES), 25-OH D_3_, cholesterol, bilirubin, and 25-OH D_2_ were purchased from Sigma Aldrich. Vitamin D_3_ recombinant rabbit monoclonal antibody (Ab) (RMH04) was purchased from Invitrogen. Vitamin D_3_ supplementation tablet was purchased from Naturevia.

### Characterization techniques

Fourier transform infrared spectroscopy (FT-IR) spectrum of NGA was acquired on the Nicolet iS5 instrument (Thermo Scientific) equipped with a ZnSe attenuated total reflectance (ATR) accessory by dropping an aqueous dispersion of the material onto the face of the ZnSe crystal. After the dispersion dried off, the ATR-corrected spectrum was collected by summing up 32 scans in the range of 500‒4000 cm^‒1^ with a resolution of 4 cm^‒1^. Baseline corrections were applied to the spectrum. X-ray photoelectron spectroscopy (XPS) measurements were conducted using a Nexsa G2 spectrometer (Thermo Fisher Scientific) with an Al Kα radiation source. The acquired data were processed and analyzed using Avantage software.

### Synthesis of nitrogen-doped graphene acid

NGA was synthesized according to the procedure described previously [39]. Briefly, 5 g of graphite, fluorinated (polymer, Sigma Aldrich), was dispersed in 300 mL *N,N*-dimethylformamide (DMF, for peptide synthesis, Sigma Aldrich). The dispersion was sonicated for 4 h and stirred overnight. Then, 15 g of sodium azide (NaN_3_, BioXtra, Sigma Aldrich) was added to the mixture, which was heated at 130 °C for 3 days. Then, the material was filtered and washed using DMF, acetone, ethanol, water, acidic water, and water till the conductivity of the filtrate was below 100 μS cm^‒1^. Then, the purified pellet was dispersed in aqueous 45 wt. % nitric acid and refluxed for 1 day at 100 °C. After that, the material was purified by filtration and subsequent washing using water, acetone, ethanol, and water. The material was then dialyzed for 3 weeks for further purification. Optionally, the material was freeze-dried.

### Microscopic and spectroscopic techniques

High-resolution transmission electron microscopy (HR-TEM) images were obtained using a FEI HR-TEM TITAN 60–300 microscope with an extreme field emission gun (X-FEG) type emission gun, operating at 300 kV. Scanning transmission electron microscopy high-angle annular dark-field imaging (STEM-HAADF) analysis for energy-dispersive X-ray spectroscopy (EDS) elemental mapping on the products was performed with a Titan HR-TEM microscope operating at 80 kV. For this analysis, a droplet of an aqueous dispersion of the material under study with a concentration of ≈0.1 mg mL^–1^ was deposited on a carbon-coated copper grid and dried at room temperature for 24 h. Scanning electron microscopy (SEM) images were obtained by a Jeol-7900F SEM microscope, with an accelerating voltage of 5.0 kV.

### Electrochemical measurement

The electrochemistry experiment was performed using a Metrohm µAutolab III (MetrohmAutolab B.V., Netherlands) controlled by the NOVA software package (version 2.1.4) with screen-printed electrodes (C110, Dropsens) in a three-electrode system: a carbon electrode was used as a counter electrode, an Ag/AgCl as a reference electrode, and a modified carbon as a working electrode. The impedance.py package [42] was used to calculate the charge transfer resistance (*R*_CT_) from the experimental data of the Nyquist plot in the equivalent Randles circuit (Figure S1). All experiments were conducted in 10 mmol L^‒1^ PBS (pH 7.4), 137 mmol L^‒1^ NaCl electrolyte, and 1 mmol L^‒1^ [Fe(CN)_6_]^3−/4‒^ served as a redox probe. The electrochemical impedance spectroscopy (EIS) measurement was conducted at a frequency range from 10 kHz to 0.1 Hz, with an applied potential of 0.12 V and an amplitude of 10 mV.

### Modification of SPCE electrode with NGA

Prior to the modification of SPCE electrodes, the stock solution of NGA (2 mg mL^‒1^ in ddH_2_O) was sonicated 10 min at 30 W and then centrifuged at 14,000 rpm for 10 min to remove aggregates and obtain the finest sheets of NGA for modification of WE. The modification of working electrodes (WE) with NGA was performed as follows: 6 μL of NGA (2 mg mL^‒1^) was drop-cast four times on the surface of WE, allowed to dry after individual drop-casts, and subsequently tested via EIS at ambient laboratory temperature (20 ± 2 °C).

### Preparation of immunosensor

All immobilization steps were performed in a humid chamber to prevent the evaporation of the modifying solution on WE electrodes. Initially, the carboxyl groups on the SPCE/NGA electrodes were activated using EDC/NHS chemistry [43, 44] to enable covalent binding of anti-25(OH)D_3_ antibody. The WE electrode was coated with 10 µL of EDC/NHS solution (50/50 mmol L^‒1^) in 100 mmol L^‒1^ MES buffer (pH 5.6) for 45 min, followed by a 10-s wash in PBS and drying. Then, 10 µL of anti-25(OH)D_3_ antibody solution (10 µg mL^‒1^ in PBS) was uniformly dispersed on the activated SPCE/NGA electrode for 1 h. The SPCE/NGA/Ab electrodes were subsequently washed with PBS for 10 s to remove the unbound antibodies and dried. To block nonspecific binding sites on the SPCE/NGA/Ab electrodes, 10 µL of BSA solution (0.25% w/v in PBS) was drop-cast onto the electrode surface for 1 h. Finally, the SPCE/NGA/Ab/BSA electrodes were thoroughly washed in PBS, dried, and stored at 4 °C until use.

The coupling efficiency of EDC/NHS was optimized using two concentrations: 50/50 mmol L^‒1^ and 100/100 mmol L^‒1^. The 10 µL of EDC/NHS solution in 100 mmol L^‒1^ MES buffer (pH 5.6) was dropped onto the WE electrode for 45 min, followed by wash in PBS. The modified SPCE-NGA/Ab electrodes were then evaluated in PBS in the presence of [Fe(CN)_6_]^3−/4‒^ redox probe. The blocking efficiency of bovine serum albumin (BSA) was evaluated at three concentrations: 0.25%, 2%, and 10% (w/v) in PBS. A 10 µL aliquot of each BSA solution was drop-cast onto the SPCE-NGA electrodes, followed by incubation for 1 h at room temperature. Subsequently, the electrodes were washed three times with PBS and characterized in PBS containing redox probe. Finally, the concentration and the incubation time of anti-25(OH)D_3_ antibody used for the detection of 25-OH D_3_ were optimized using EIS measurements in the presence of redox probe. A 10 µL aliquot of anti-25(OH)D_3_ antibody solution at the defined concentration (25, 10 and 2.5 µg mL^-1^) was dispersed onto an activated SPCE/NGA electrode for 1 h. The electrodes were subsequently washed with PBS for 10 s and dried. To block nonspecific interactions, 10 µL of BSA solution (0.25% w/v in PBS) was drop-cast onto the electrode surface and incubated for 1 h. The SPCE/NGA/Ab/BSA electrodes were then thoroughly washed with PBS and characterized. The incubation time was optimized for an antibody concentration of 10 µg mL^-1^ and times of 1, 2 and 3 hours.

### Sensing of 25-OH vitamin D_3_

For the construction of calibration curve, 10 µL of different concentrations of 25-OH D_3_ in PBS (10 mmol L^−1^, pH 7.4), i.e., 0.5 ng mL^‒1^, 5 ng mL^‒1^, 10 ng mL^‒1^, 50 ng mL^‒1^, 100 ng mL^‒1^, and 500 ng mL^‒1^ was prepared and incubated on SPCE/NGA/Ab/BSA electrodes for 1 h. After a washing step, EIS measurements were performed. The calibration curve was fitted with a 4-parameter logistic regression (1):

1 $$y = \frac{{A}_{1}-{A}_{2}}{1+{\left(\frac{x}{{x}_{0}}\right)}^{P}}+{A}_{2}$$

where *y* is the electrochemical signal (*R*_CT_), *A*_1_ and *A*_2_ are the bottom and upper asymptotes of the sigmoidal curve, *x* is the 25-OH D_3_ concentration, *x*_0_ represents the concentration at half-maximum of the curve (EC_50_), and *p* represents the slope at the inflection point. The working ranges were evaluated as intervals between EC_20_ and EC_80_.

The LOD was estimated from the regression curves as the concentrations corresponding to the sum of the background value obtained by the logistic fit (*A*_1_) and three times the standard deviation of the blank (*s*_0_):

2 $$y\left(\mathrm{LOD}\right)=A_1+3s_0$$

The sensitivity of the immunosensor was calculated from the slope (*p*) of the regression curve and the geometric area of the WE electrode (*A*):

3 $$\mathrm{sensitivity}=p/A$$

### Real serum analysis

Human serum sample was employed to evaluate the performance of the constructed immunosensor for 25-OH D_3_ detection. A serum sample from a healthy volunteer at the Faculty Hospital in Olomouc was collected and stored at 4 °C until analysis. The serum sample was used undiluted and spiked with 25-OH D_3_ at two concentrations, 20 and 40 ng mL^–1^, within the range of the calibration curve. Both spiked and non-spiked samples were analyzed using the proposed immunosensor.

### Specificity and stability of immunosensor

The specificity of the immunosensor towards 25-OH D_3_ was evaluated by EIS measurement of its analog, vitamin D_3_ (100 ng mL^‒1^), cholesterol (7 mmol L^−1^), bilirubin (20 mmol L^−1^), and 25-OH D_2_ (100 ng mL^‒1^). A vitamin D_3_ supplement tablet (commercial product composed of calcium phosphate, magnesium stearate, microcrystalline cellulose, cholecalciferol 1000 I.U./tablet) was crushed into a powder, and vitamin D_3_ was dissolved in absolute ethanol. The solution was centrifuged to remove solid particles, and the supernatant containing vitamin D_3_ at a concentration of 25 µg mL^‒1^ was diluted with PBS to achieve a final concentration of 100 ng mL^‒1^. To assess the stability of the SPCE/NGA/Ab/BSA immunosensor, electrodes were dried in air and maintained in a parafilm-sealed petri dish with silicate beads at 4 °C for 30 days. EIS response was measured consecutively after 5 days.

## Results and discussion

### Structural, morphological, and (electro)chemical characterization of NGA

**Fig. 1:**
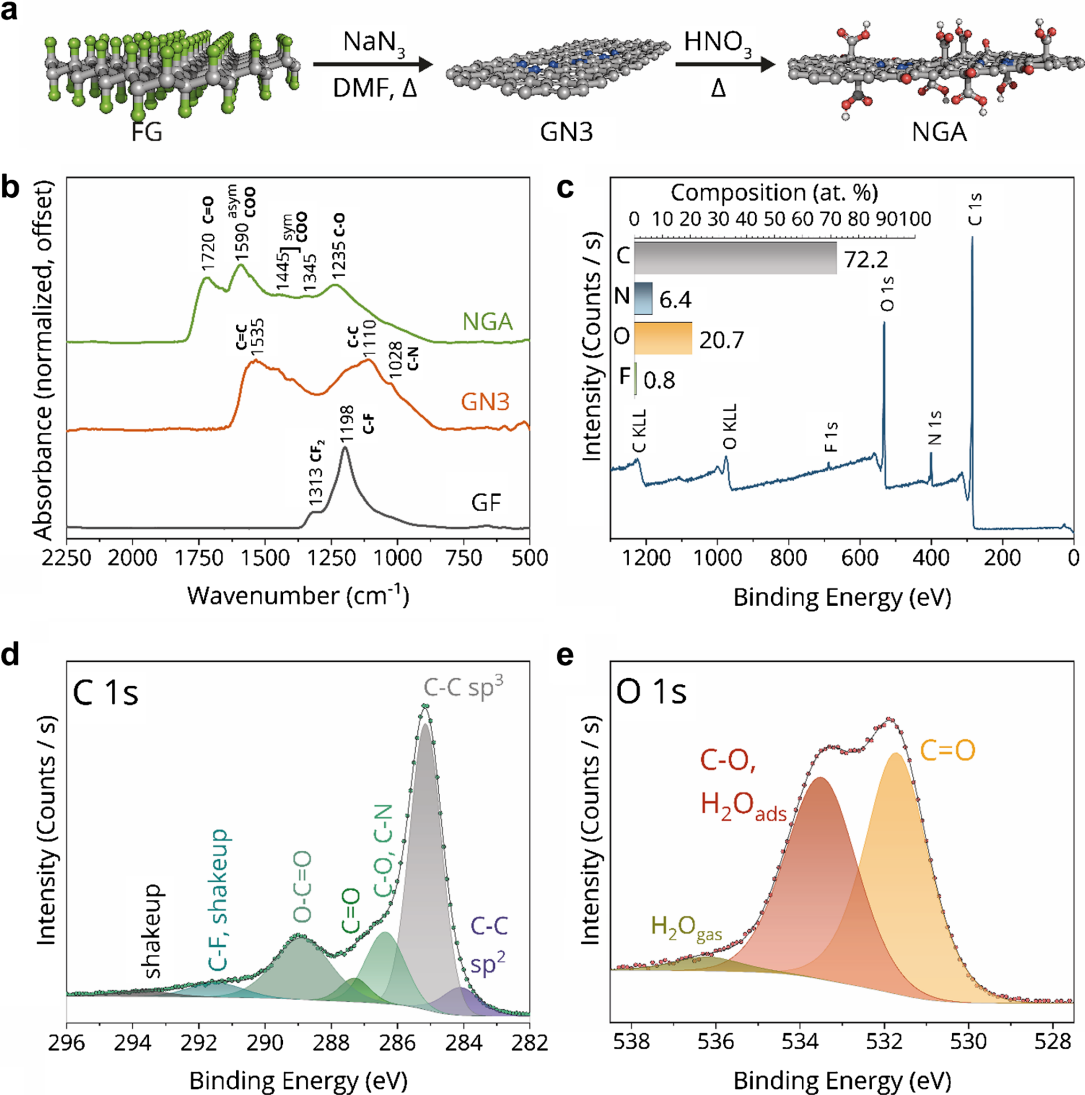
**a** Schematic representation of NGA synthesis; **b** FT-IR spectra of the graphite fluoride (GF) parent material, nitrogen-doped graphene intermediate (GN3), and final nitrogen-doped graphene acid (NGA) used in the study; **c** survey XPS spectrum of the NGA material with its elemental composition in the inset, deconvoluted C 1 s **d** and O 1 s **e** XPS spectral regions of NGA

The synthesis of the NGA material was performed using a wet-chemistry approach starting from fluorographene, as illustrated in Fig. 1a. Initially, graphite fluoride was dispersed in dimethylformamide (DMF) and subjected to sonication to yield a fluorographene dispersion. This dispersion was subsequently reacted with sodium azide at 130 °C, producing GN3, a nitrogen-doped graphene derivative [45]. Following filtration and washing, the GN3 intermediate was oxidized using 45 wt.% aqueous nitric acid. The resulting NGA material was recovered by filtration and subjected to further purification via dialysis of its aqueous dispersion.

The synthesis process was monitored using FT-IR (Fig. 1b). After the initial reaction of fluorographene with sodium azide, the loss of fluorine during the reaction was evident from the disappearance of characteristic C − F and CF_2_ vibrational modes at 1198 and 1313 cm^−1^, respectively. This translated into the formation of a defective sp^2^-conjugated network, distinctive of broad features centered at 1535 and 1110 cm^−1^ [38], characteristic of carbonyl groups [38, 39]. The presence of carboxyl groups was further evidenced by their fingerprint bands at 1590, 1445, 1345, and 1230 cm^−1^, corresponding to asymmetric and two symmetric COO⁻ stretching modes and C − O stretching mode, respectively [39].

Raman spectroscopy was employed to further characterize NGA (Fig. S2). The spectrum exhibits the characteristic D (≈1345 cm^−1^) and G (≈1590 cm^−1^) bands, with an intensity ratio (*I*_*D*_/*I*_*G*_) of 1.28. This relatively high ratio reflects a substantial defect density arising from extensive carboxyl functionalization [46]. The pronounced D band further indicates disruption of the graphene lattice, generating abundant defect sites and edge functionalities that are advantageous for biosensing applications.

XPS unraveled the chemical composition of the NGA material. The survey spectrum (Fig. 1c) revealed an almost complete removal of fluorine (0.8 at.%) and confirmed nitrogen doping (6.4 at.%) along with a high content of oxygen-containing functional groups (20.7 at.%) with no significant contamination by other elements. Analysis of the C 1 s spectral region (Fig. 1d) indicated that only a small fraction (7.1%) of carbon atoms existed within sp^2^ graphitic domains at 284.1 eV. This suggests that the oxidation process disrupted most of the sp^2^-conjugated network, introducing substantial defects. The majority of carbon atoms (50.2%) were present in sp^3^ hybridized states, reflecting the high functionalization and defect density of the material. However, due to the complexity of distinguishing sp^2^ from sp^3^ carbons in mixed hybridization systems, these values should be interpreted cautiously [47].

A significant fraction of carbon atoms (19.8%) was found as carboxylic groups at 288.9 eV, [38, 39, 48], while the component at 285.9 eV (15.6%) suggested contributions from both nitrogen doping and adventitious oxygen bound to carbon. A small number of carbon groups was associated with lone carbonyl groups (4%) observed at 287.3 eV. Additionally, the peak at 291.2 eV, associated with C − F bonds, overlaps with the shake-up satellite of carbon, complicating the quantification of the C − F bond component. Deconvolution of the O 1 s spectral region (Fig. 1e) revealed three main components. Most oxygen atoms were associated with carbonyl groups (50.4%). It is worth noting that the highly hydrophilic nature of NGA likely resulted in an overestimation of oxygen atoms in the C − O region due to adsorbed water molecules. This was supported by the presence of a component at 536.2 eV, attributed to water vapor above the material’s surface, consistent with previous reports [49, 50]. Detailed deconvolution parameters are provided in the Supporting Information (Tables S1 and S2).

**Fig. 2:**
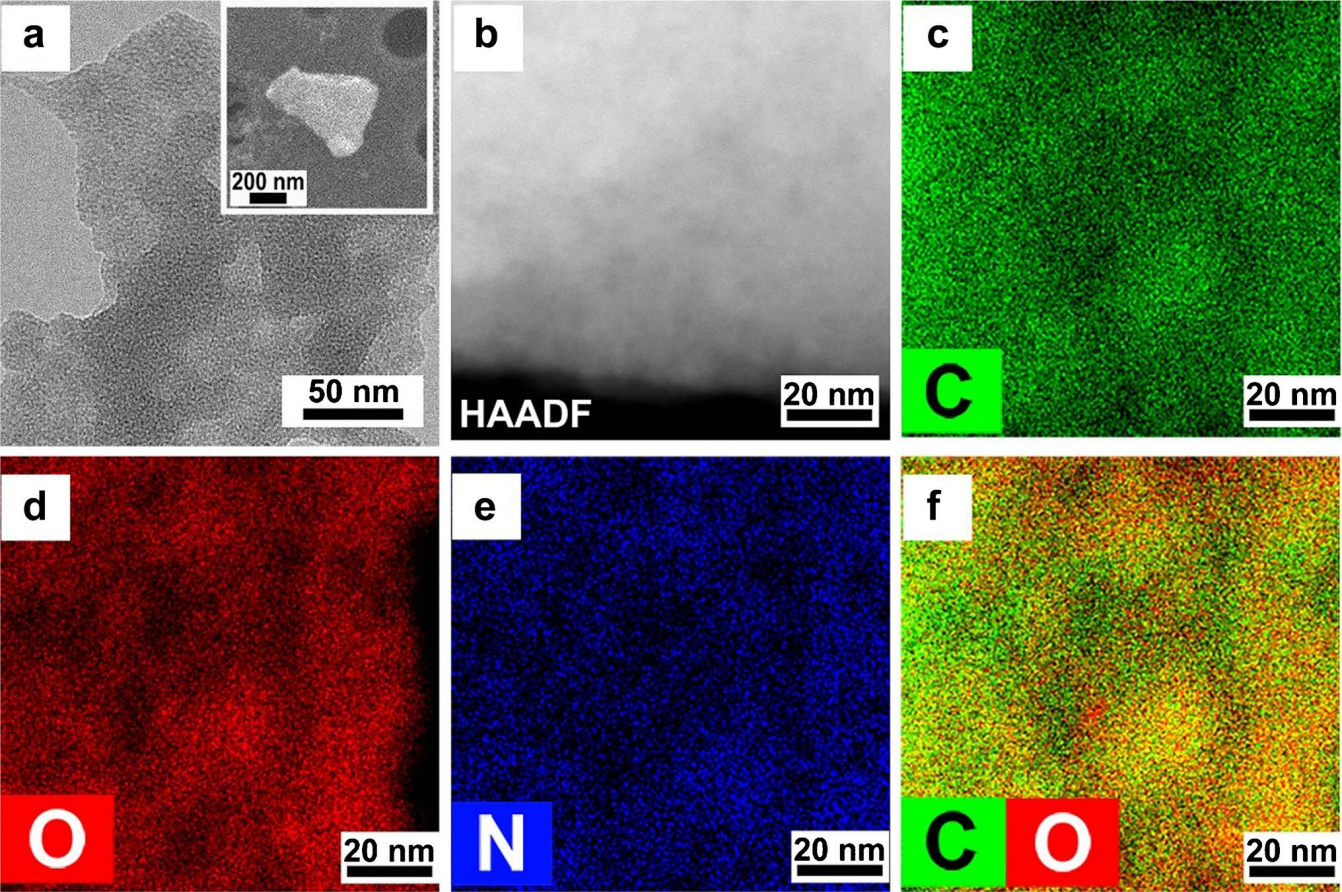
**a** HR-TEM image of NGA flake (inset: SEM image of NGA). **b** HAADF-STEM image of NGA flake. EDS chemical mapping of NGA for **c** carbon, **d** oxygen, and **e** nitrogen. **f** Combined chemical mapping of carbon and oxygen

 HR-TEM and SEM analyses showed that the NGA consists of a few layered graphene flakes with a lateral size of around 600 nm (Fig. 2a, b and SEM image with lateral size in the inset). Chemical mapping of the NGA confirmed the homogeneous distribution of oxygen and nitrogen elements on the surface of individual NGA flakes, which is consistent with the spatial distribution of carbon (Fig. 2c).

### Electrode modification with NGA

At the initial stage of immunosensor development, we compared the electrochemical responses of graphene acid (GA) and nitrogen-doped graphene acid (NGA) using EIS and CV (Fig. S3). NGA consistently exhibited enhanced electrochemical performance relative to GA, with EIS providing the clearest distinction between the two materials. The superior performance of NGA can be explained by the synergistic effect of nitrogen doping on the graphene framework. First, the incorporation of nitrogen heteroatoms increases the density of electroactive sites and improves charge delocalization within the sp^2^ lattice, thereby facilitating faster electron transfer. Second, nitrogen functionalities enhance the hydrophilicity and chemical reactivity of the material, leading to better colloidal stability in aqueous environments and more efficient covalent coupling of antibodies through available carboxyl groups. In addition, the uniform distribution of carboxyl groups in NGA provides a chemically defined and reproducible platform for controlled bioconjugation, minimizing variability in antibody orientation and density. Building on these results, we further examined the influence of successive NGA depositions on the electrode surface using EIS. The corresponding Nyquist plots (Fig. S4a) illustrate the electrochemical changes observed across four consecutive drop-casting steps of NGA onto SPCEs. After the first deposition, the *R*_CT_ remained comparable to that of the bare SPCE, suggesting incomplete surface coverage. From the second deposition onward, however, a progressive decrease in charge transfer resistance was observed, reaching its lowest value after the fourth drop-casting step (Fig. S4b). This trend indicates that multiple depositions are required to achieve a uniform and conductive NGA layer that maximizes electron transfer efficiency. The observed decrease in *R*_CT_ with increasing NGA layers can be explained by percolation theory, as previously reported for MWCNT/epoxy nanocomposite biosensors [51]. Nitrogen doping, the presence of sp^2^-hybridized carbon atoms, and the assembly of graphene sheets near the percolation threshold collectively enhance the formation of electroactive sites between the NGA sheets, thereby facilitating electron transfer and reducing charge transfer resistance. These findings suggest that a single drop-casting round was insufficient to fully cover the working electrode with nanomaterial. However, from the second round onward, the electrode surface became uniformly coated with NGA, leading to improved electron transfer. For all subsequent electrochemical measurements, the fourth drop-casting round (≈0.6 µg cm^−2^ of NGA) was selected as the optimal modification step.

### Optimization of antibody immobilization

**Fig. 3:**
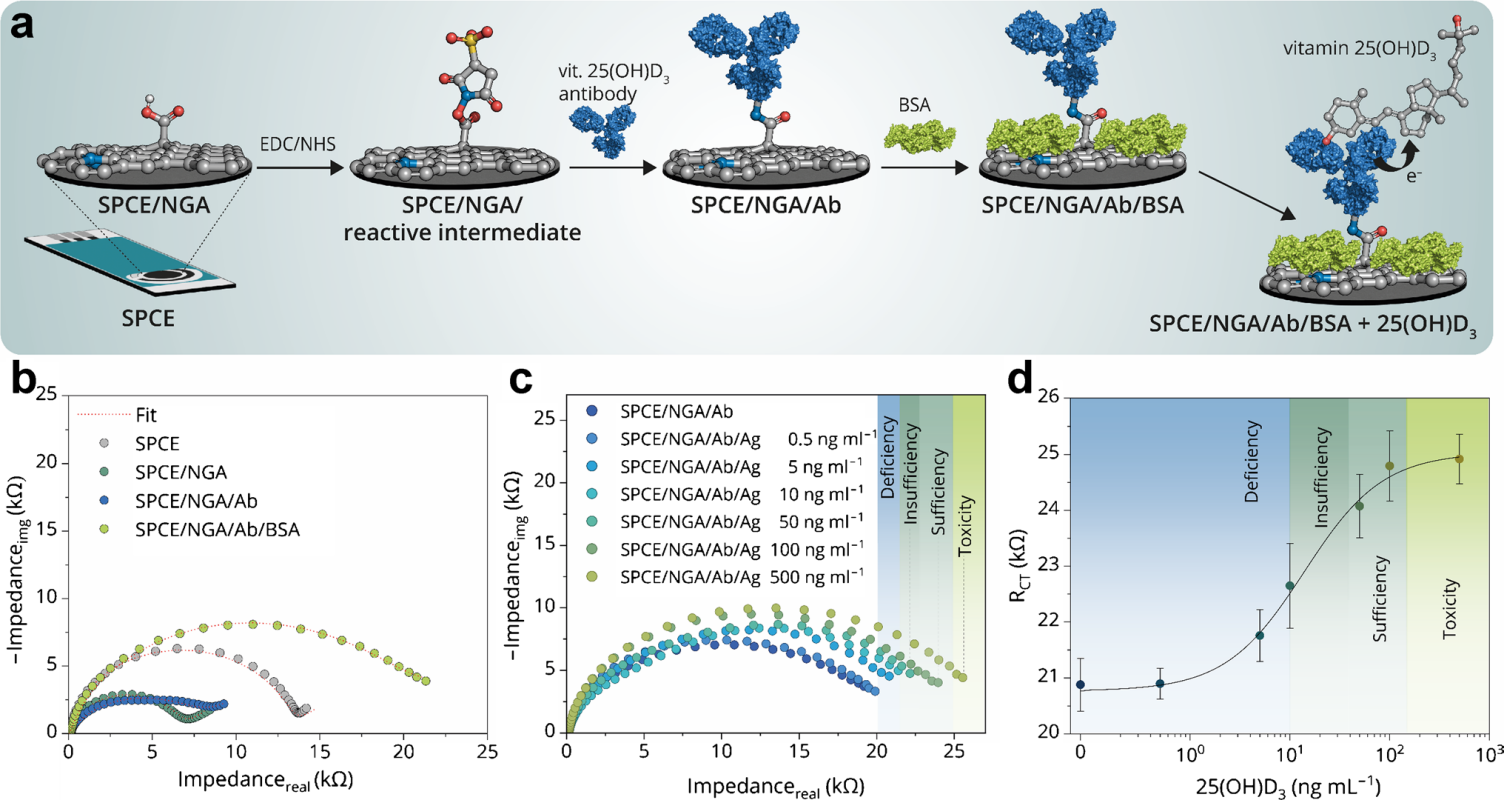
**a** Schematic representation of the immunosensor construction process. **b** Nyquist plot showing impedance changes after antibody immobilization (10 mg mL^‒1^) on the SPCE/NGA/Ab electrode. The plot also illustrates the subsequent impedance response following BSA adsorption on the modified electrode. **c** Nyquist plots demonstrating the impedance response of the SPCE/NGA/Ab immunosensor at varying concentrations of 25-OH D_3_. **d** Calibration curve of the impedimetric immunosensor (*R*_CT_ vs. log c), constructed based on impedance measurements. All measurements were performed in the presence of [Fe(CN)_6_]^3‒/4‒^ as a redox probe. Statistical analysis was conducted using SPCE (*n* = 5), and results are presented as mean ± standard deviation.

Electrochemical measurements using a redox probe were further employed to assess the binding of anti-25(OH) vitamin D₃ antibodies onto EDC/NHS-activated NGA sheets. The concentration of EDC/NHS was optimized by testing two different ratios (Fig. S5). No significant differences were observed in *R*_CT_ values between the 50/50 and 100/100 mmol L^‒1^, indicating that carboxyl groups were fully activated and accessible for antibody coupling in both concentrations. The concentration of bovine serum albumin used for surface blocking was optimized by testing three different concentrations (Fig. S6). Our results demonstrated that 0.25% (w/v) BSA was sufficient to effectively block the surface of SPCE-NGA electrodes and that the higher concentrations commonly employed in ELISA assays (1–3%) were not required to prevent nonspecific interactions. Finally, the effects of antibody concentration and incubation time on covalent bonding between the antibody’s amino groups and the activated carboxyl groups of NGA were evaluated, both in the presence and absence of the BSA blocking agent (Fig. 3 and Fig. S7, S8).

Interestingly, a decrease in *R*_CT_ was observed after antibody immobilization on the NGA-modified electrodes, as shown in Fig. S7. This effect can be attributed to the electrostatic properties of the antibody molecules [52, 53]. The anti-25(OH)D_3_ antibodies, which carry a net positive charge under the experimental conditions, likely mask the negatively charged carboxyl groups of NGA. As a result, the local surface potential is altered, enhancing electrostatic attraction of the negatively charged [Fe(CN)_6_]^3‒/4‒^ redox probe toward the electrode surface [54]. Improved accessibility of the redox probe facilitates electron transfer and consequently reduces the overall charge transfer resistance. Similar effects have been reported in other impedimetric immunosensor systems, where the charge distribution of immobilized biomolecules strongly influenced the impedance response [55]. To experimentally verify this hypothesis, we attempted to measure the ζ-potential of the modified electrode surfaces. However, reliable values could not be obtained because NGA removed from SPCE surface produced scattering signals below the detection threshold of the instrument. Despite this limitation, the electrostatic explanation remains the most plausible interpretation of the observed behavior and is supported by comparable findings in related EIS-based biosensing studies.

Once the SPCE/NGA/Ab surface was blocked with BSA, charge transfer resistance increased (Fig. 3 and Fig. S8). This rise in *R*_CT_ can be attributed to the overall blocking of the SPCE/NGA/Ab surface by BSA, as well as its negative charge repelling the negatively charged [Fe(CN)_6_]^3‒/4‒^ redox probe. Antibody concentrations higher than 0.5 µg mL^‒1^ did not significantly alter *R*_CT_ values, suggesting that all available antibody binding sites on the NGA-activated surface had been utilized. Similarly, time-dependent incubation studies revealed that incubation times longer than 1 h did not significantly increase *R*_CT_ values, indicating that shorter incubation periods are sufficient for effective covalent immobilization of antibodies on NGA sheets (Fig. S8).

### Detection of 25-OH vitamin D₃

Following the optimization of NGA deposition, antibody concentration, and incubation time, the SPCE/NGA/Ab immunosensor was constructed and employed for the electrochemical detection of 25-OH D_3_, as shown in Fig. 3a‒d. The sensor was tested across a concentration range of 0.5‒500 ng mL^‒1^, encompassing the reference values for vitamin D_3_ in human serum [4].

The antigen–antibody interaction was reflected in an increase in charge transfer resistance with rising 25-OH D_3_ concentrations, as observed in the Nyquist plot (Fig. 3b). The impedance spectra exhibited a characteristic semicircular shape, indicative of electron charge transport without the presence of Warburg impedance, which is typically associated with diffusion-controlled processes. The raw impedance data were fitted using a modified Randles circuit (Fig. S1), and the extracted *R*_CT_ values were plotted to construct a calibration curve (*R*_CT_ vs. log c) (Fig. 3c, d).

The proposed immunosensor demonstrated a limit of detection (LOD) of 1.49 ng mL^‒1^ (3.72 µM) and a sensitivity of 1.97 kΩ ng^‒1^ mL cm^‒2^, corresponding with a value of 5.08 10^‒4^ ng Ω^‒1^. The working range, determined using a four-parameter logistic regression model as the interval between EC_20_ and EC_80_, was 3.96‒48.83 ng mL^‒1^, covering the physiologically relevant vitamin D_3_ concentrations, from deficiency to optimal levels. The linearity of calibration curve and corresponding calibration curve equation is shown in Fig. S9. Given that the optimal serum level of 25-OH D_3_ ranges between 30 and 100 ng mL^‒1^, and insufficiency is defined as 20‒30 ng mL^‒1^, the sensor effectively operates within the critical range for clinical diagnostics [56]. It is worth noting that the achieved operational range and LOD are comparable with traditional methods used in analytical laboratories including, for instance, high-performance liquid chromatography (HPLC, LoQ 3 ng mL^‒1^) [57]. Also, the reported immunosensors have achieved comparable or superior sensitivity and detection limits; for instance, ATP@AuNPs/RGO-SeO_2_ on SPCE [58] achieved an impressive detection limit of 0.01 ng mL^‒1^ with a working range of 0.05‒200 ng mL^‒1^. Similarly, Asp-Gd_2_O_3_NRs on ITO [34] reported a detection limit of 0.1 ng mL^‒1^ with a working range of 10–100 ng mL^‒1^. Additional examples supporting these comparisons are summarized in Table S3. However, these approaches rely on multiple nanomaterials, including heavy metals, which increase fabrication costs and reduce sustainability. In contrast, our constructed immunosensor provides a simple, cost-effective, and metal-free alternative with high sensitivity. The working range is well-suited for practical applications, as it specifically covers the clinically relevant vitamin D_3_ concentration range. Additionally, NGA offers chemically well-defined, conductive, and sustainable 2D material composed of biogenic elements. It is affordable, easy to synthesize, and does not require additional surface modification for biomolecule immobilization. The strategic positioning and abundance of carboxyl groups on the NGA surface enable controllable covalent immobilization of biorecognition elements without significantly compromising conductivity, ensuring both high sensitivity and reproducibility of the immunosensor.

### Real serum analysis

**Fig. 4:**
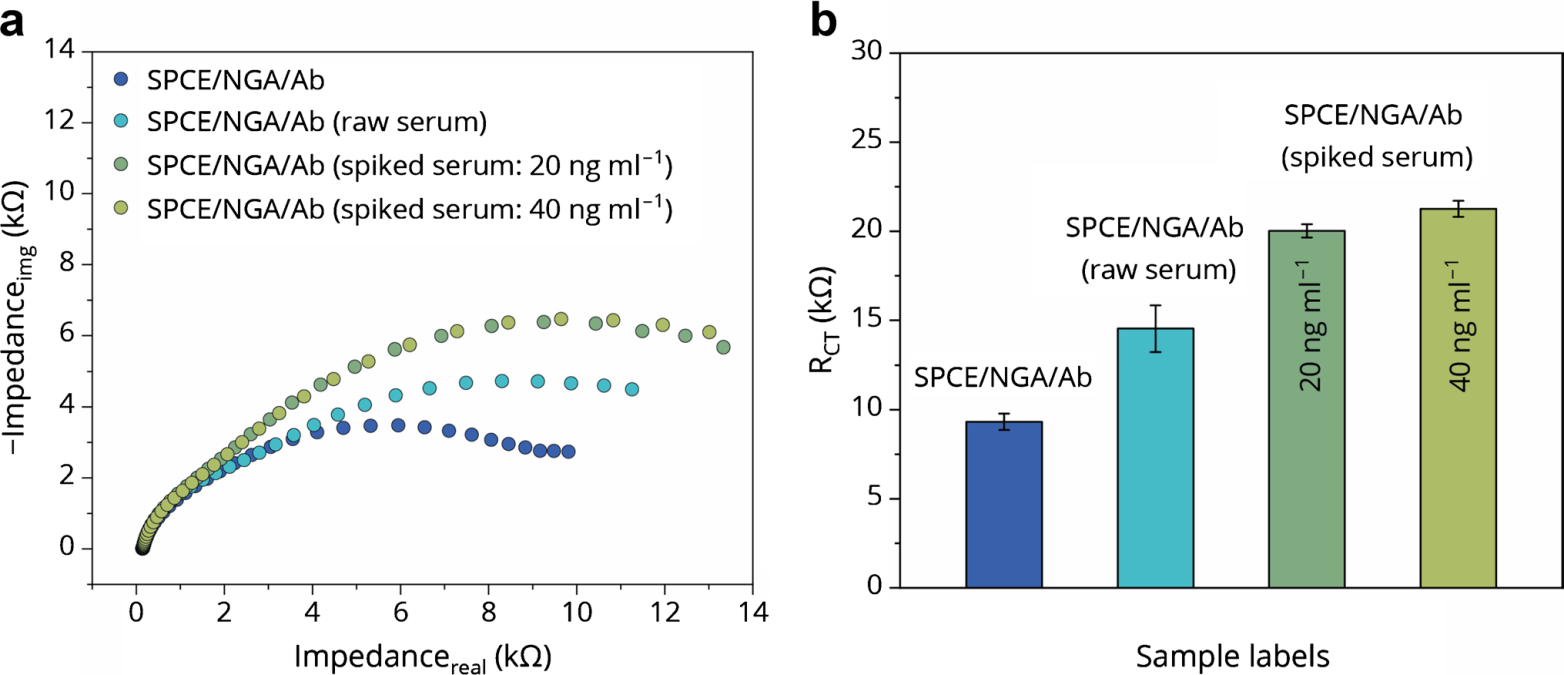
The Nyquist plot **a** and column graph **b** of applicability of proposed immunosensor in real human serum

To evaluate the applicability of the designed immunosensor for real sample analysis, we tested both native human serum and serum samples spiked with 25-OH D_3_. Real biological samples frequently impair assay performance due to the inherent complexity of the serum matrix. In addition, the majority of vitamin D metabolites are bound to vitamin D binding protein (DBP) and, to a lesser extent, to albumin. As a result, the immunosensor primarily interacts with and detects the DBP–25(OH)D_3_ complex rather than the free vitamin form. This was reflected by increased Δ*R*_CT_ values of approximately 5 kΩ and 6.5 kΩ for concentrations of 20 and 40 ng mL^–1^, respectively—about twice as high as the Δ*R*_CT_ responses obtained for 25-OH D_3_ in PBS (Fig. 4a, b). Importantly, the preliminary serum data revealed trends consistent with those observed in PBS, thereby confirming the sensor’s capability to recognize the target analyte within a complex biological matrix.

### Cross-reactivity and storage stability of the immunosensor

#### Cross-reactivity evaluation

**Fig. 5:**
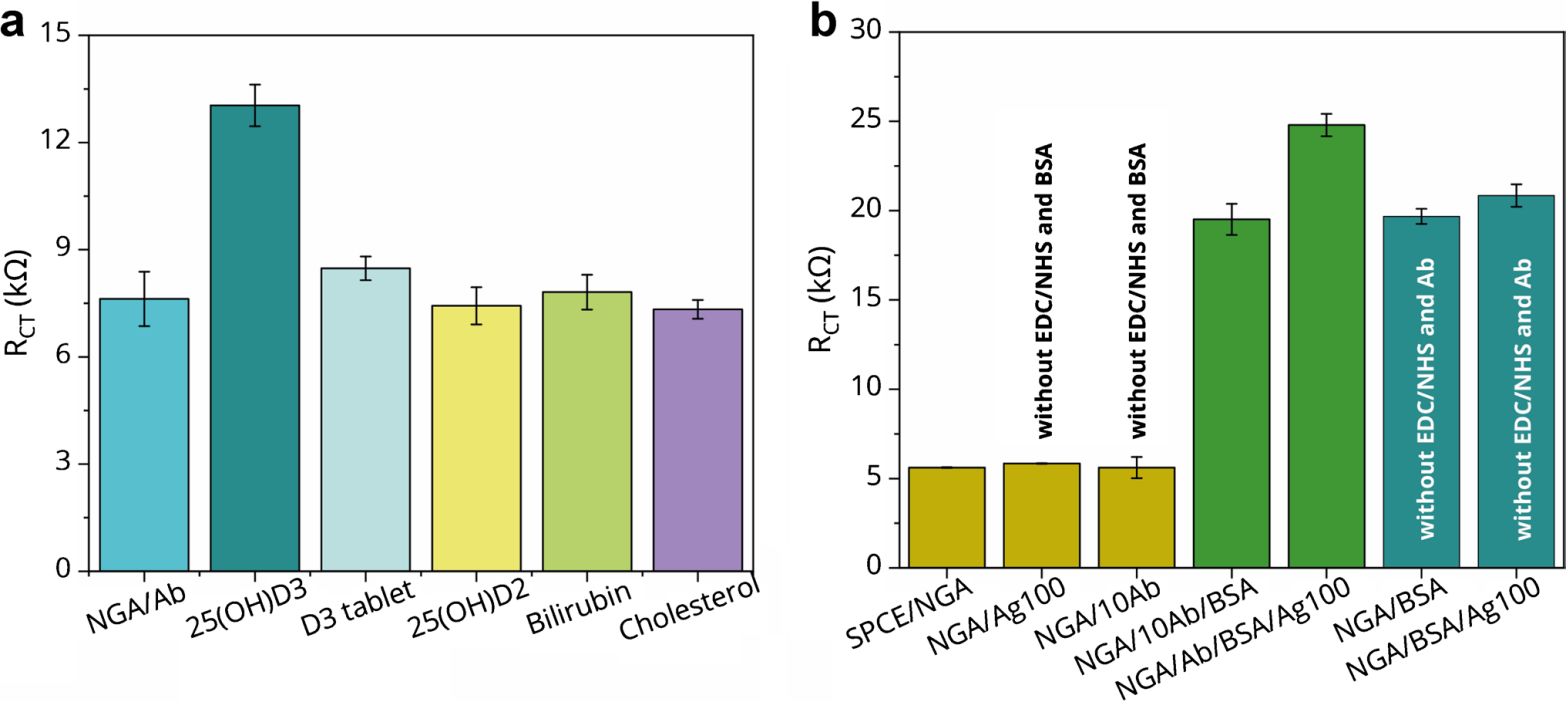
Cross-reactivity of the SPCE/NGA/Ab immunosensor. **a** Selectivity of the immunosensor against common blood interferents, including cholesterol, bilirubin, and vitamin D_2_. **b** Dark yellow columns show the adsorption of 25-OH D_3_ antigen (100 ng mL^−1^; 100 Ag) and antibodies (10 μg mL^−1^) on the SPCE/NGA surface. Green columns represent the non-specific interaction of 25-OH D_3_ with the SPCE/NGA/BSA surface in the presence of 100 ng mL^−1^ antigen. Teal columns indicate the impedance response arising from non-specific adsorption between BSA and 25-OH D_3_. Statistical analysis was performed using SPCE (*n* = 4), and data are expressed as mean ± standard deviation

To evaluate the specificity of the immunosensor toward 25-OH D_3_, SPCE/NGA/Ab electrodes were examined for potential non-specific interactions. Two sets of control experiments were performed. In the first, selected blood-derived interferents—cholesterol, bilirubin, 25-OH D_2_, and a vitamin D_3_ analog—were tested, as shown in Fig. 5a. No significant responses were observed relative to the control sample, confirming the antibody’s high specificity for the 25-OH D_3_ form. The second control series assessed potential adsorption of molecules on differently modified electrode surfaces. As shown in Fig. 5b (dark yellow columns), no appreciable non-specific interactions were detected when 25-OH D_3_ antigen (100 ng mL^−1^; 100 Ag) and antibody (10 µg mL^−1^; 10 Ab) were applied to SPCE/NGA electrodes lacking surface activation and BSA passivation. To further explore the possibility of matrix-related effects, electrodes modified with SPCE/NGA/BSA were exposed to 100 ng mL^−1^ of the antigen (Fig. 5b; green columns). The impedance response remained nearly unchanged compared with the background signal, indicating minimal non-specific binding of 25-OH D_3_ to the passivated surface. The teal columns in Fig. 5b represent the impedance response associated with possible non-specific adsorption between BSA and 25-OH D_3_. The signal was negligible and comparable to the baseline, demonstrating that the blocking layer effectively prevents unintended interactions with the antigen. Taken together, these findings confirm that the developed immunosensor provides highly specific recognition of 25-OH D_3_, with negligible interference from structurally related molecules or non-specific adsorption processes.

#### Storage stability assessment

**Fig. 6:**
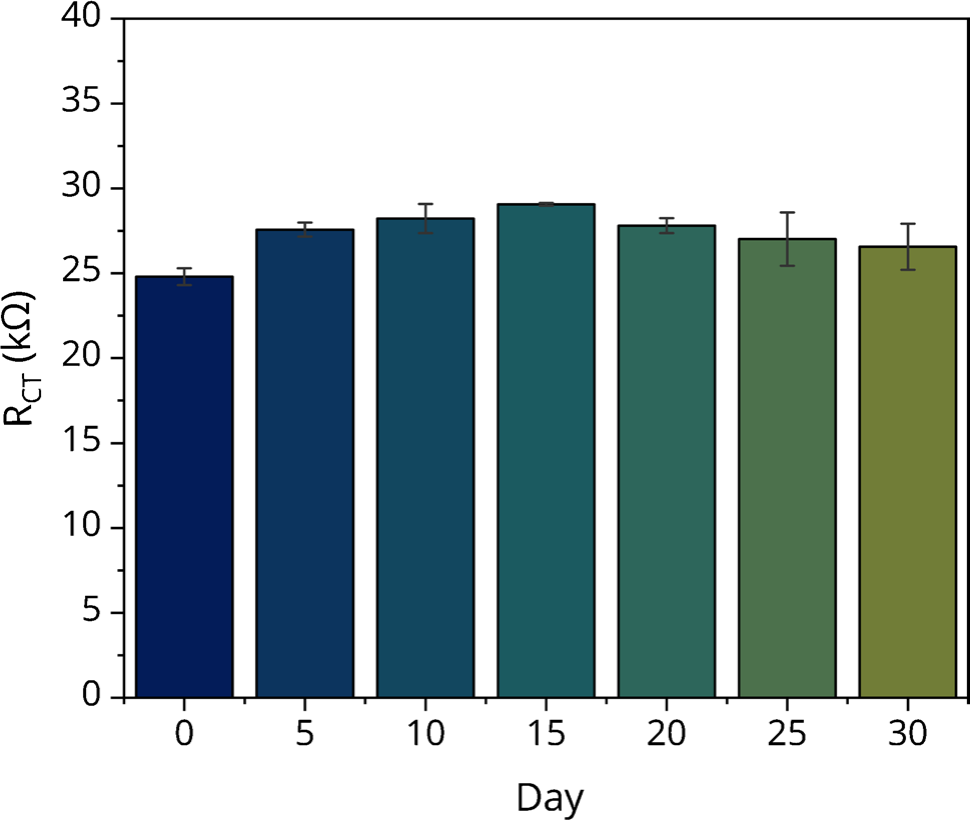
Storage stability of the SPCE/NGA/Ab immunosensor. Impedance response of the immunosensor after storage at 4 °C. The stability of the sensor was assessed over a defined period, showing consistent performance. Statistical analysis was conducted using SPCE (*n* = 4), and results are presented as mean ± standard deviation

To evaluate the storage stability of the SPCE/NGA/Ab immunosensor, dry electrodes were stored at 4 °C in a refrigerator and tested periodically over 30 days. Impedance measurements were performed every 5 days, with results presented in Fig. 6. An initial increase in *R*_CT_ values was observed compared to day 0, followed by a relatively stable response throughout the storage period. This early fluctuation may be attributed to surface rearrangement or reorganization of biomolecules immediately after fabrication, leading to a transient decrease in electrochemical impedance response. Despite this initial variation, the immunosensor demonstrated consistent performance over 30 days, confirming its long-term stability for practical applications.

#### Reproducibility

To assess the reproducibility of the proposed label-free immunosensor, a total of 12 electrodes were prepared under identical conditions using independent batches of SPCEs electrodes and subsequently evaluated across a range of antigen concentrations (0.5, 50, and 100 ng mL^−1^). The relative standard deviation (RSD) was calculated using the following Eq. 4:

4 $$\text{RSD(\%)}=\left(\frac{\text{SD}}{{\text{mean}}}\right)\bullet 100$$

**Fig. 7:**
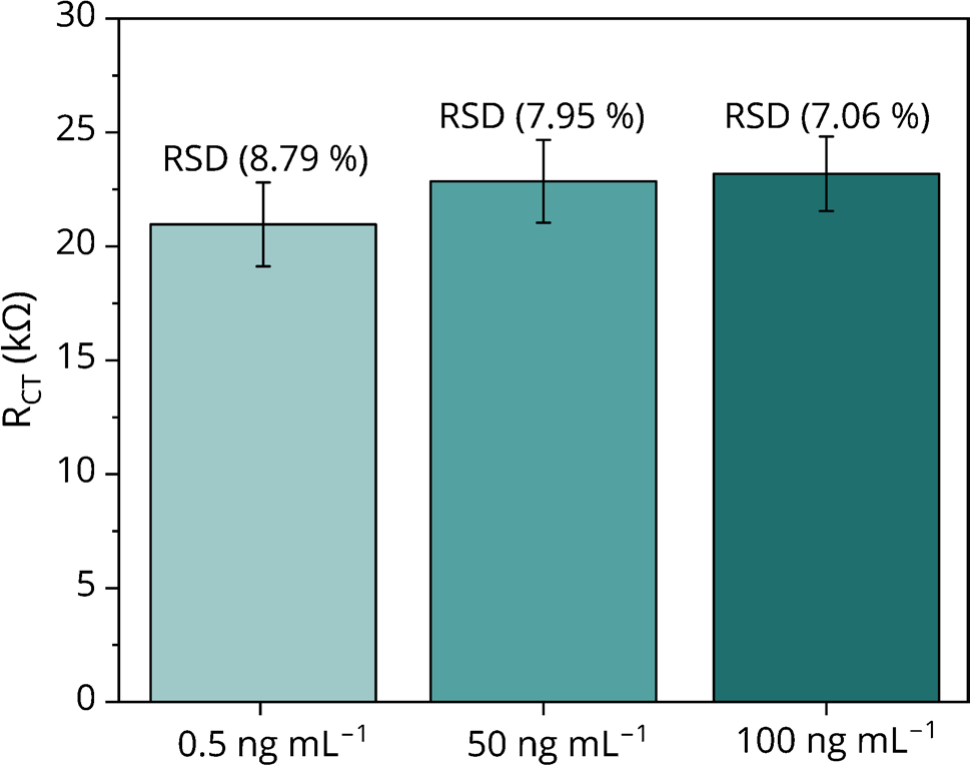
Reproducibility experiment (*n* = 12) for three concentrations of antigen expressed as relative standard deviation (RSD)

RSD obtained for 0.5, 50, and 100 ng mL^−1^ was 8.79, 7.95, and 7.06%, respectively (Fig. 7). These results demonstrate the good reproducibility of the immunosensor across different batches. The reliability of an immunosensor depends on multiple factors and the variability in electrode fabrication, antibody immobilization, or measurement conditions undermines reliability. For example, the manufacturer of SPCEs declares repeatability ≤ 5% within a batch. Thus, the electrode batches may significantly differ in responses and must be, for example, individually calibrated. Ultrasonication and drop-casting of NGA solution are two crucial factors influencing the final sensor performance, with human error during drop-casting being a major source of variability. Additionally, the reliability of purchased antibody batches remains a common challenge in the development of immunosensors.

## Conclusion

In this work, we introduced a streamlined and sustainable electrochemical immunosensor utilizing nitrogen-doped graphene acid (NGA) for the selective detection of 25-hydroxy vitamin D_3_. The strategic choice of NGA as a metal-free transducer material enabled efficient antibody immobilization through its carboxyl groups, contributing to both sensor stability and biocompatibility. The sensor’s analytical performance—characterized by a clinically relevant detection range and strong signal response—positions it as a practical tool for point-of-care diagnostics. Notably, the system retained its sensing capabilities over extended storage periods for up to 30 days, reflecting robustness and reliability in operational environments. In comparison with conventional vitamin D assays, this platform offers a more accessible and cost-effective alternative without compromising performance. The absence of metals further enhances its environmental and health safety profile. Overall, this study provides a ground for the broader application of metal-free nanomaterials in biosensor technologies, with future prospects in portable diagnostic systems and routine clinical screening.

## Supplementary information

Below is the link to the electronic supplementary material.

ESM 1 (DOCX 3.54 MB)

## Data Availability

Data are available via ZENODO repository at 10.5281/zenodo.17348052.
