# Supplementary material for: A label-free impedimetric immunosensor based on nitrogen-doped graphene acid for sensitive detection of vitamin D3
Source: Mikrochim Acta. 2025 Nov 18;192(12):823. doi: 10.1007/s00604-025-07625-9 (PMC12627205; doi:10.1007/s00604-025-07625-9)
Supplement: Supplementary file 1 — (DOCX 3.54 MB) [file 604_2025_7625_MOESM1_ESM.docx]

Supporting information

to

**A label-free impedimetric immunosensor based on nitrogen-doped graphene acid for sensitive detection of vitamin D_3_**

Jakub Janek^a^, Zdenka Fohlerova^a,b,*^, Ivan Dědek^c^, Vítězslav Hrubý^c^, David Panáček^c,d^, Jaromir Hubalek^a^, Roman Havlík^e^, Radek Zbořil^c,d^, Michal Otyepka^c,f^, Petr Jakubec^c,*^

^a^Department of Microelectronics, Faculty of Electrical Engineering and Communication, Brno University of Technology, Technická 10, 616 00 Brno, Czech Republic.

^b^Department of Biomedical Engineeering, Faculty of Electrical Engineering and Communication, Brno University of Technology, Technická 10, 616 00 Brno, Czech Republic.

^c^Regional Centre of Advanced Technologies and Materials (RCPTM), Czech Advanced Technology and Research Institute (CATRIN), Palacký University Olomouc, Šlechtitelů 27, Olomouc 783 71, Czech Republic

^d^Nanotechnology Centre, Centre for Energy and Environmental Technologies, VSB–Technical University of Ostrava, 17. listopadu 2172/15, 708 00 Ostrava-Poruba, Czech Republic

^e^Department of Surgery I, Faculty of Medicine and Dentistry, Palacký University Olomouc and University Hospital Olomouc, 771 47 Olomouc, Czech Republic

^f^IT4Innovations, VSB – Technical University of Ostrava, 17. listopadu 2172/15, Ostrava-Poruba 708 00, Czech Republic

**Scheme of Randles circuit used for fitting of EIS data**


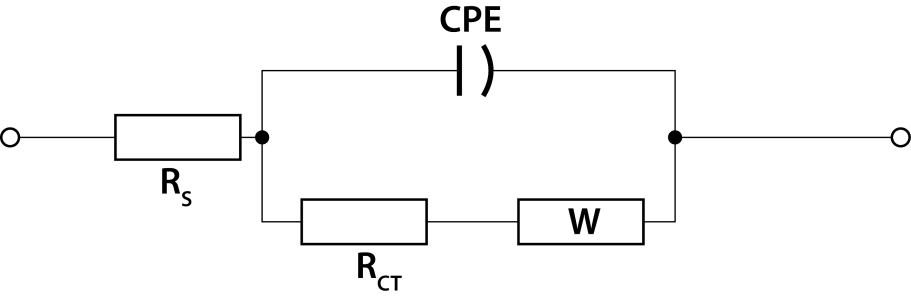


**Figure S1.** Randles equivalent circuit used to fit EIS experimental data.

**Raman spectrum**


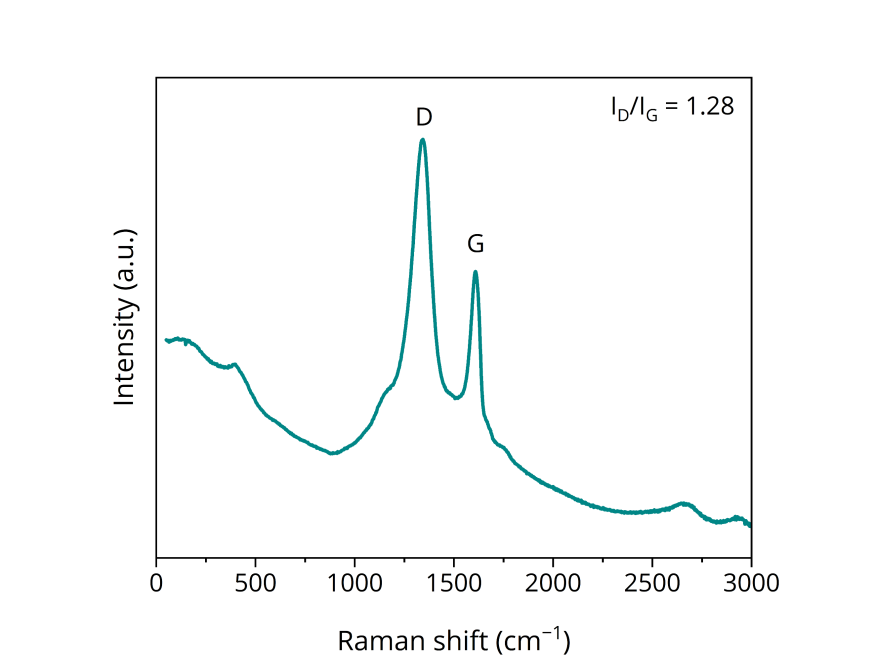


**Figure S2.** Raman spectrum of NGA

**Comparison of electrochemical signal of NGA and GA**


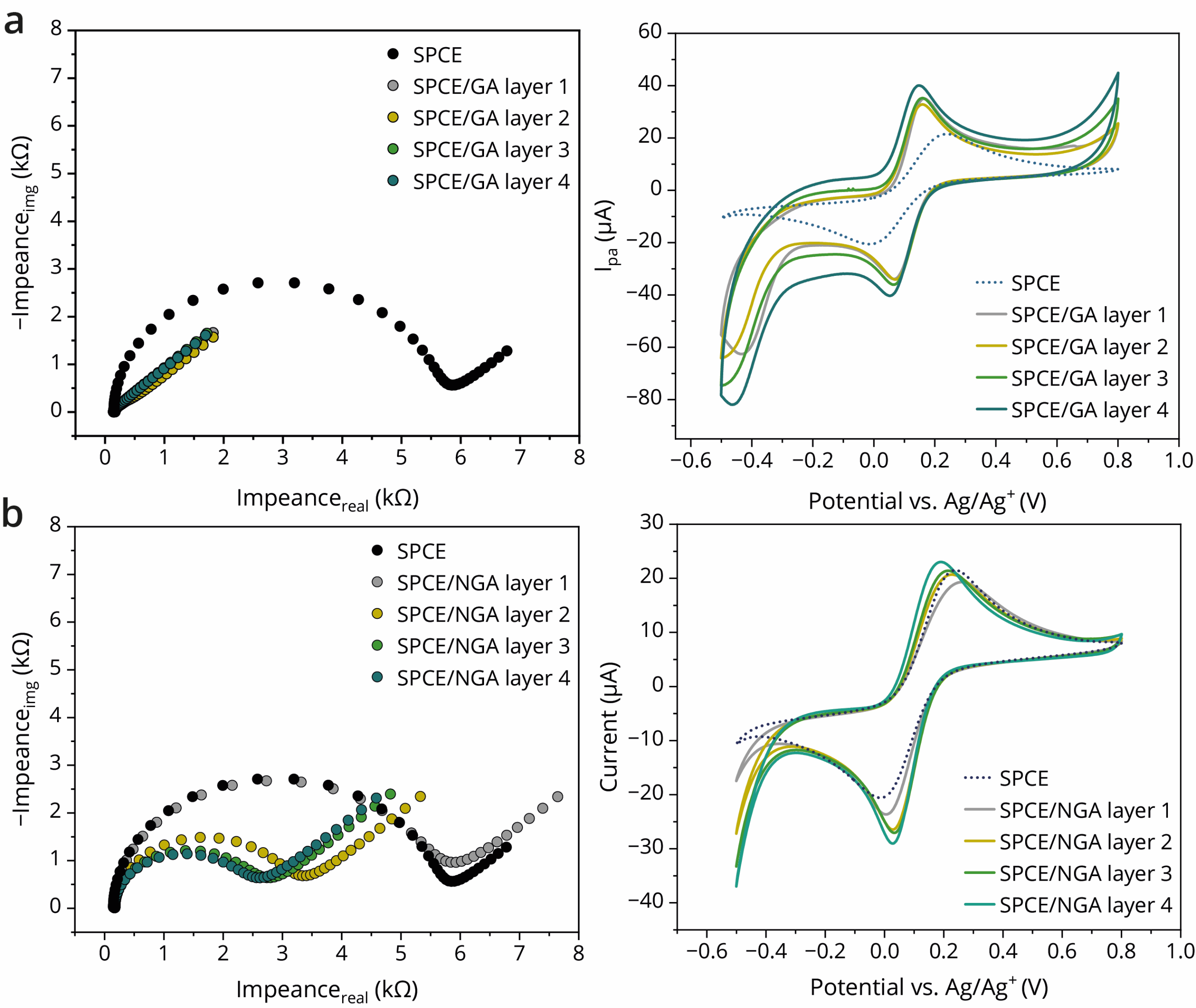


**Figure S3.** Comparison of electrochemical responses of SPCEs modified with **(a)** graphene acid (GA) and **(b)** nitrogen-doped graphene acid (NGA), evaluated by EIS and CV. The data highlight the influence of nitrogen doping on electrode performance and illustrate the differences in electrochemical behavior between GA- and NGA-modified electrodes.

**Optimization of NGA deposition on SPCE electrode**


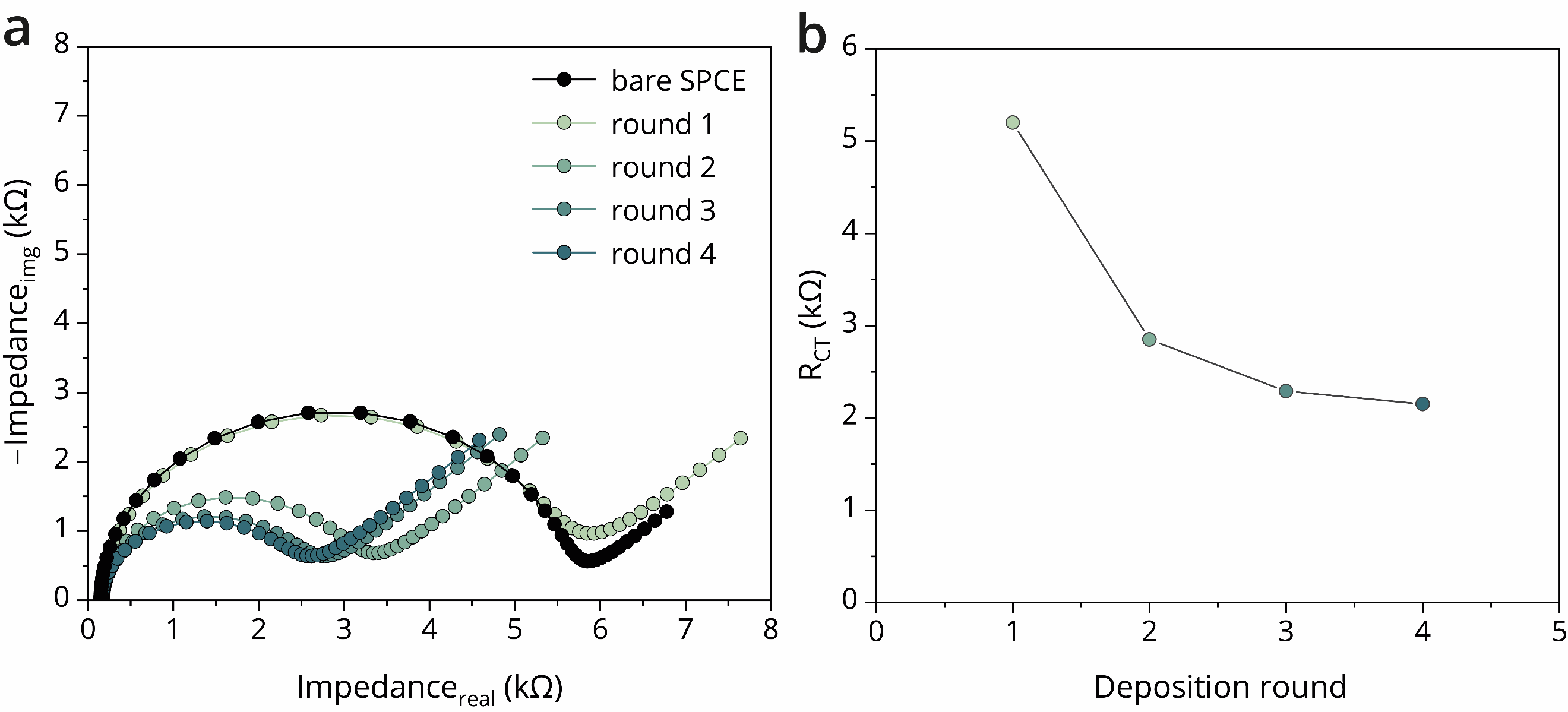


**Figure S4.** Three concentrations of anti-25(OH)D_3_ were tested: 25, 10 and 2.5 µg mL^-1^ with incubation times of 1, 2 and 3 hours for each antibody concentration. A) Nyquist plots of SPCE electrodes as a function of NGA deposition rounds. The EIS was performed in the presence of [Fe(CN)_6_]^3−/4-^ as a redox probe. B) R_CT_ vs. deposition rounds of NGA on screen printed carbon electrodes.

**Optimization of EDC/NHS concentration**


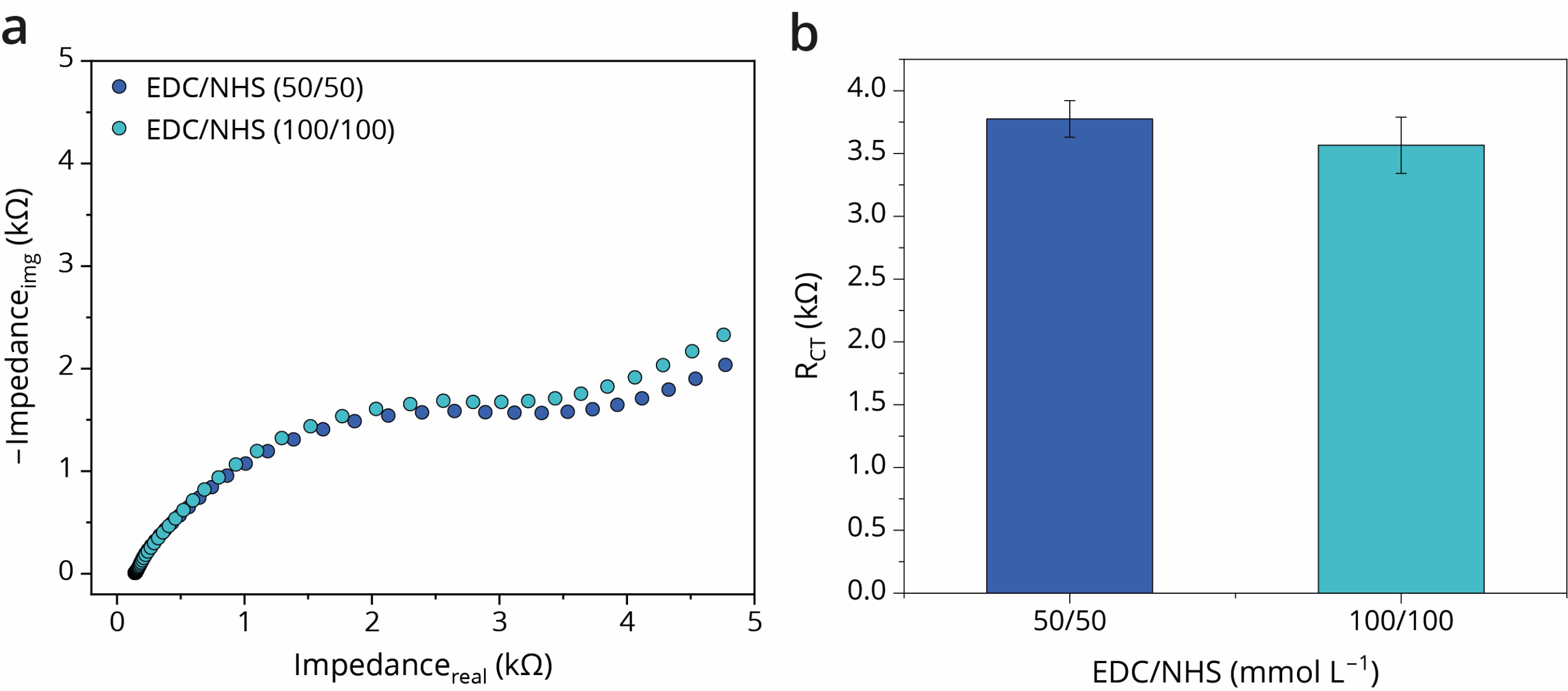


**Figure S5.** EDC/NHS optimization using two concentrations: 50/50 mmol L^‒1^ and 100/100 mmol L^‒1^. The impedance signal was obtained in the presence of redox probe after immobilization of 10 µg mL^‒1^ anti-25(OH)D_3_ antibody (n = 2).

**Optimization of BSA concentration**


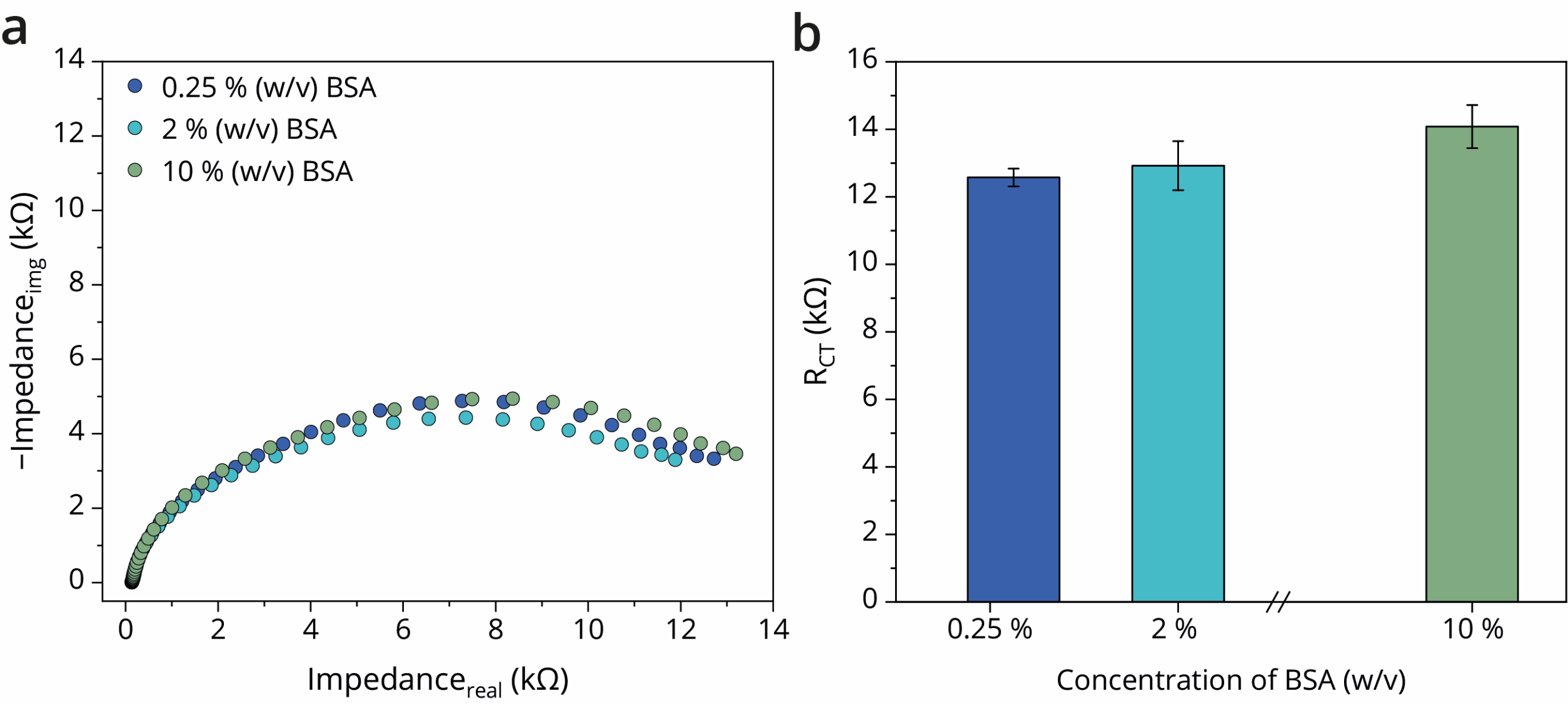


**Figure S6.** Nyquist plots of BSA optimized procedure. The column graph shows the evaluated R_CT_ values for three different concentrations of BSA adsorbed on SPCE-NGA electrodes (n = 2).

**Optimization of antibody immobilization**

**
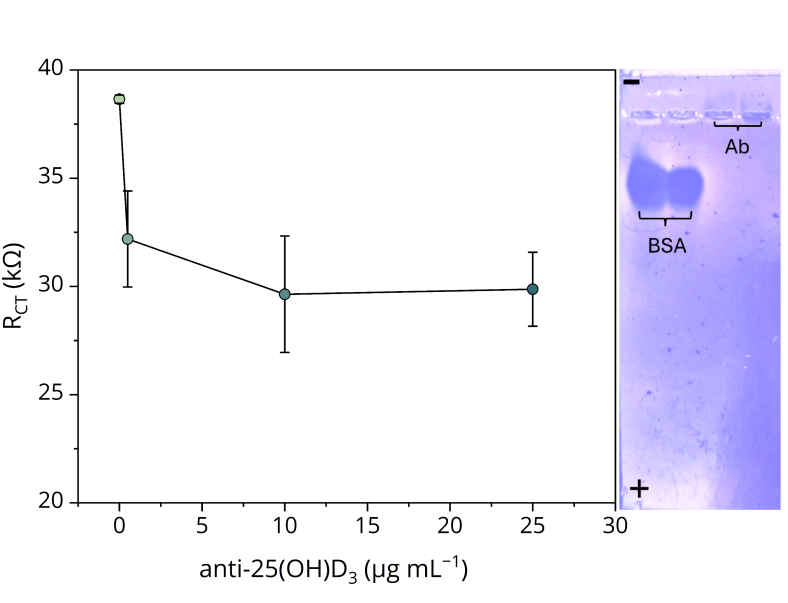
**

**Figure S7.** EIS responses SPCE-NGA electrodes to 10 µg mL^-1^ to antibody after 3 hours of incubation, without performing BSA blocking step and image illustrating electrophoresis of BSA and antibody at pH 7.4.


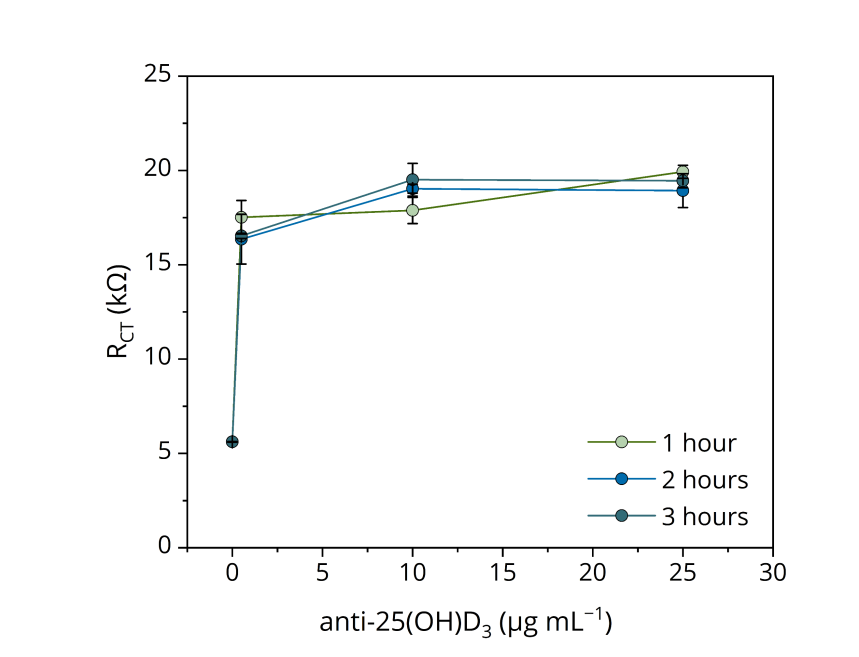


**Figure S8.** Optimization of antibody concentration and incubation time for immobilization of antibody on SPCE-NGA electrode using EDC/NHS chemistry and a BSA blocking step. Measurements were performed in the presence of [Fe(CN)_6_]^3−/4−^ as a redox probe. Statistical analysis was performed with n = 4, and the results are expressed as mean ± standard deviation.


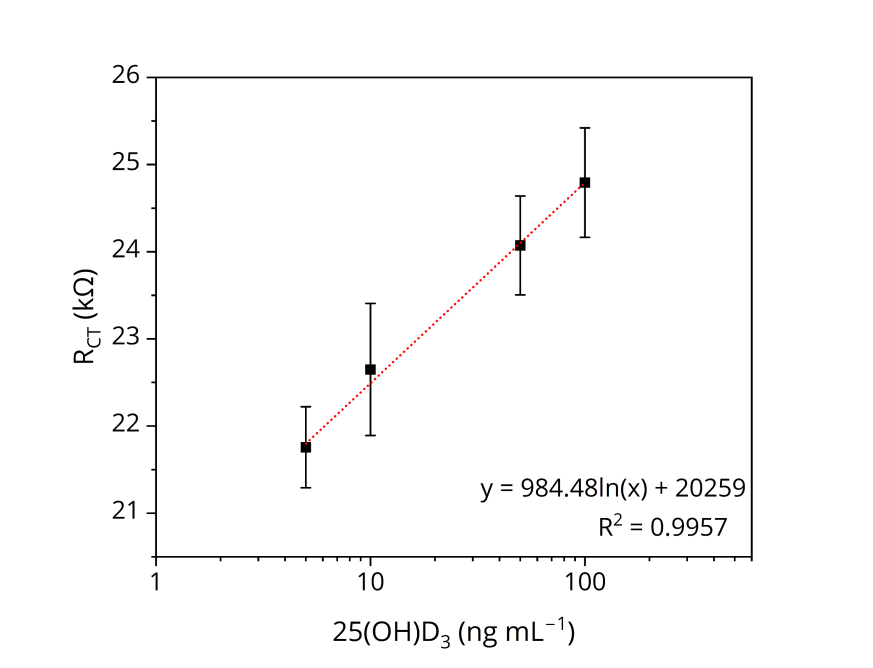


**Figure S9**. Linear range of calibration curve and corresponding curve equation.

**Table S1.** Deconvolution parameters for the C 1s XPS spectral line of NGA material.

| **Component** | **Peak BE (eV)** | **FWHM (eV)** | **Area (%)** |
| --- | --- | --- | --- |
| **C-C sp^2^** | 284.1 | 1.3 | 6.1 |
| **C-C sp^3^** | 285.2 | 1.1 | 50.2 |
| **C-O/C-N** | 286.4 | 1.4 | 15.6 |
| **C=O** | 287.3 | 1.1 | 4.0 |
| **O=C-O** | 288.9 | 2.0 | 19.8 |
| **C-F** | 291.5 | 2.0 | 4.4 |

**Table S2.** Deconvolution parameters for the O 1s XPS spectral line of NGA material.

| **Component** | **Peak BE (eV)** | **FWHM (eV)** | **Area (%)** |
| --- | --- | --- | --- |
| **C=O** | 531.7 | 1.8 | 50.4 |
| **C-O, H_2_O_ads_** | 533.5 | 2.0 | 46.4 |
| **H_2_O_gas_** | 536.2 | 1.9 | 3.2 |

**Table S3.** Summary table of analytical performances of 25-OHD_3_ electrochemical immunosensors

| **Sensing materials** | **Detection techniques** | **Linear range**  **(ng mL^‒1^)** | **Detection limit**  **(ng mL^‒1^)** | **Sensitivity** | **Ref** |
| --- | --- | --- | --- | --- | --- |
| AuNPs/RGO-SeO2/SPE | EIS | 0.05–200 ng mL^‒1^ | 0.01 ng mL^‒1^ | n/a | [S1] |
| BSA/Ab-VD/Asp-Gd_2_O_3_NRs/ITO | DPV | 0–100 ng mL^‒1^ | 0.10 ng mL^‒1^ | 0.38 μA ng^−1^ mL cm^−2^ | [S2] |
| Ag-25(OH)D_3_/BSA/ Ab-25(OH)D_3_-Fc/GNRs/GCE | DPV | 1–100 ng mL^‒1^ | 0.1 ng mL^‒1^ | n/a | [S3] |
| Apt/MoS2/ErGO/GCE | DPV | 0.1–150 ng mL^‒1^ | 0.02 ng mL^‒1^ | n/a | [S4] |
| LaNPs-GQDs/ZIF-8/GCE | DPV | 2.5−500 ng mL^‒1^ | 2.44 ng mL^‒1^ | n/a | [S5] |
| Ag-Ag_2_ O-CNT/GCE | EIS | 8–40 ng mL^‒1^ | 3.16 ng mL^‒1^ | 0.0147 nM^−1^ cm^−2^ | [S6] |
| GCE/CuCo_2_O_4_/NCNTs/  P-GO/25(OH)D_3_-imprinted  PPy | DPV | 0.8–4010 ng mL^‒1^ | 0.152 ng mL^‒1^ | n/a | [S7] |
| BSA/AB-25VitD_3_/CS@GdNPs/ITO | DPV | 1–100 ng mL^‒1^ | 4.5 ng mL^‒1^ | 5.99 μA ng^−1^ ml cm^−2^ | [S8] |
| BSA/anti-25VD_3_/nCeO2/CC | DPV | 1–200 ng mL^‒1^ | 4.63 ng mL^‒1^ | 2.08 μA ng^−1^ mL cm^−2^ | [S9] |
| CYM@AuMNPs/SPE | DPV | 7.4–70 ng mL^‒1^ | 2.4 ng mL^‒1^ | 0.55 μA ml ng^−1^ cm^‒2^ | [S10] |
| **BSA/AB/NGA/SPCE** | **EIS** | **3.96‒48.83 ng mL^‒1^** | **1.49 ng mL^‒1^** | **1.97 kΩ ng^‒1^ mL cm^‒2^** | **Present work** |

S1. Magar HS, Brahman PK, Hassan RYA (2022) Disposable impedimetric nano-immunochips for the early and rapid diagnosis of Vitamin-D deficiency. Biosens Bioelectron X 10:100124. https://doi.org/10.1016/j.biosx.2022.100124

S2. Chauhan D, Kumar R, Panda AK, Solanki PR (2019) An efficient electrochemical biosensor for Vitamin-D3 detection based on aspartic acid functionalized gadolinium oxide nanorods. J Mater Res Technol 8:5490–5503. https://doi.org/10.1016/j.jmrt.2019.09.017

S3. Anusha T, Bhavani KS, Hassan RYA, Brahman PK (2023) Ferrocene tagged primary antibody generates electrochemical signal: An electrochemical immunosensing platform for the monitoring of vitamin D deficiency in clinical samples. Int J Biol Macromol 239:124269. https://doi.org/10.1016/j.ijbiomac.2023.124269

S4. Park J, Kim M, Kim W, et al (2022) Ultrasensitive detection of 25-hydroxy vitamin D3 in real saliva using sandwich-type electrochemical aptasensor. Sens Actuators B Chem 355:131239. https://doi.org/10.1016/j.snb.2021.131239

S5. Anusha T, Bhavani KS, Shanmukha Kumar JV, Brahman PK (2021) Synthesis and characterization of novel lanthanum nanoparticles-graphene quantum dots coupled with zeolitic imidazolate framework and its electrochemical sensing application towards vitamin D3 deficiency. Colloids Surf Physicochem Eng Asp 611:125854. https://doi.org/10.1016/j.colsurfa.2020.125854

S6. Sana Fathima TK, Ramaprabhu S (2023) An Antibody-Free, Silver-Silver Oxide-Carbon Nanotube Nanocomposite-Based Impedimetric Sensor for the Nanomolar Detection of 25-Hydroxyvitamin D_3_. J Electrochem Soc 170:087513. https://doi.org/10.1149/1945-7111/acef5c

S7. Sheikh Beig Goharrizi MA, Kazemi Oskuee R, Aleyaghoob G, et al (2023) A new molecularly imprinted polymer electrochemical sensor based on CuCo_2_ O_4_ /N‐doped CNTs/P‐doped GO nanocomposite for detection of 25‐hydroxyvitamin D_3_ in serum samples. Biotechnol Appl Biochem 70:357–373. https://doi.org/10.1002/bab.2363

S8. Chauhan D, Hashmi Z, Raj R, Solanki PR (2024) Microfluidic Nanobioplatform-Based Immunosensor for Monitoring of 25-Hydroxy Vitamin-D_3_. ECS Sens Plus 3:041601. https://doi.org/10.1149/2754-2726/ad9935

S9. Chauhan D, Yadav AK, Solanki PR (2021) Carbon cloth-based immunosensor for detection of 25-hydroxy vitamin D3. Microchim Acta 188:145. https://doi.org/10.1007/s00604-021-04751-y

S10. Polli F, D’Agostino C, Zumpano R, et al (2023) ASu@MNPs-based electrochemical immunosensor for vitamin D3 serum samples analysis. Talanta 251:123755. https://doi.org/10.1016/j.talanta.2022.123755
